# Cross-Sectional Time Series Analysis of Associations between Education and Girl Child Marriage in Bangladesh, India, Nepal and Pakistan, 1991-2011

**DOI:** 10.1371/journal.pone.0106210

**Published:** 2014-09-09

**Authors:** Anita Raj, Lotus McDougal, Jay G. Silverman, Melanie L. A. Rusch

**Affiliations:** 1 Division of Global Public Health, Department of Medicine, University of California San Diego School of Medicine, San Diego, CA, United States of America; 2 Center on Gender Equity and Health, University of California San Diego, San Diego, CA, United States of America; 3 Boston University School of Medicine/Boston Medical Center, Department of Medicine, Section of General Internal Medicine, Clinical Addiction Research and Education, Boston, MA, United States of America; 4 Vancouver Island Health Authority, Vancouver, Canada; Rollins School of Public Health, United States of America

## Abstract

**Background:**

Girl education is believed to be the best means of reducing girl child marriage (marriage <18 years) globally. However, in South Asia, where the majority of girl child marriages occur, substantial improvements in girl education have not corresponded to equivalent reductions in child marriage. This study examines the levels of education associated with female age at marriage over the previous 20 years across four South Asian nations with high rates (>20%) of girl child marriage- Bangladesh, India, Nepal and Pakistan.

**Methods:**

Cross-sectional time series analyses were conducted on Demographic and Health Surveys (DHS) from 1991 to 2011 in the four focal nations. Analyses were restricted to ever-married women aged 20–24 years. Multinomial logistic regression models were used to assess the effect of highest level of education received (none, primary, secondary or higher) on age at marriage (<14, 14–15, 16–17, 18 and older).

**Results:**

In Bangladesh and Pakistan, primary education was not protective against girl child marriage; in Nepal, it was protective against marriage at <14 years (AOR = 0.42) but not for older adolescents. Secondary education was protective across minor age at marriage categories in Bangladesh (<14 years AOR = 0.10; 14–15 years AOR = .25; 16–17 years AOR = 0.64) and Nepal (<14 years AOR = 0.21; 14–15 years AOR = 0.25; 16–17 years AOR = 0.57), but protective against marriage of only younger adolescents in Pakistan (<14 years AOR = 0.19; 14–15 years AOR = 0.23). In India, primary and secondary education were respectively protective across all age at marriage categories (<14 years AOR = 0.34, AOR = 0.05; 14–15 years AOR = 0.52, AOR = 0.20; 16–17 years AOR = 0.71, AOR = 0.48).

**Conclusion:**

Primary education is likely insufficient to reduce girl child marriage in South Asia, outside of India. Secondary education may be a better protective strategy against this practice for the region, but may be less effective for prevention of marriage among older relative to younger adolescents.

## Introduction

UNICEF estimates that 10 million girls marry before age 18 years annually [1], increasing their risk for maternal and child morbidities and mortality [2], [3]. Elimination of girl child marriage is believed to be key to reducing global maternal and infant mortality rates [2], [3]. Education has been identified as the single most important factor associated with girl child marriage globally [3]–[9], and major national and global efforts, including recent high-level commitments from the US State Department and UNFPA, are aimed at reducing girl child marriage via improved girl education [4]–[8]. However, in the context of South Asia, the region where half of all girl child marriages occur [1], dramatic expansions of basic education for girls over the past 20 years [10] have not corresponded with significant reductions in girl child marriage [11]. In fact, in Bangladesh and Nepal, where gender parity in education has been achieved [10], [12], [13], there has been no reduction in marriage of 16–17 year olds over the past 20 years [11].

South Asia has long contended with girl child marriage, despite establishing laws against the practice as early as 1929. Though legal age of marriage for girls was 12 at that time [14], today, most governments in the region have established 18 years as the legal age for marriage for girls. Pakistan’s law designates 16 years as the legal marital age for girls. These age-at-marriage laws are atypically enforced in the region, as marriage is viewed as a family rather than civil matter, governed by religion or culture rather than law [14]. Girls in tribal, rural and impoverished communities are most vulnerable, but variations in prevalence of the practice by subnational region and religious and ethnic group suggest the relevance of culture and history in sustaining the practice, as well [15]. Hence, girl child marriage remains socially entrenched, particularly in areas where social and gender development lag. Families arranging girls’ marriages for social and economic reasons do not always explicitly obtain her consent for the marriage [14]. Education may help support girls’ capacity to express her marital preferences, and support parents’ recognition of options for girls beyond marriage. To clarify the association between education and early marriage in the region, this study examines the associations of primary and secondary education with risk for marriage of girls across early and later adolescence over the past 20 years for the four South Asian nations most affected by this practice- Bangladesh, India, Pakistan, and Nepal.

## Methods

### Data Source

Demographic and Health Surveys (DHS) are nationally representative household surveys that measure population demographics and health indicators in low and middle income nations. Data for the current study were drawn from DHS administered to women of reproductive age (15–49 years) in Bangladesh, India, Nepal and Pakistan during the period of 1991 to 2011, in the form of independent cross-sectional studies. Survey response rates for DHS included in the current study were 94–99% [11]. For comparability of data, standardized measures and protocols are used across nations and time points. DHS protocols include standardized training and monitoring of interviewers, quality assurance reviews for data collection and entry, and safety and confidentiality procedures. Oral informed consent for the interview/survey was obtained from all respondents by interviewers. Further details on these procedures are available elsewhere [16]–[28].

### Ethics statement

All DHS procedures were reviewed and approved by nation-specific ethical review boards and the institutional review board (IRB) of ORC Macro in the United States. Current analyses were reviewed and approved by the University of California, San Diego IRB.

### Sampling plan

DHS utilize stratified cluster randomized sampling procedures [29], [30]. Stratification is conducted by rural and urban areas and country-specific geographic or administrative regions. Random clusters of households are selected from each stratified area using population census data. Within each cluster, approximately 25 households are selected for inclusion in the study based on equal probability systematic sampling. Within each selected household, one eligible woman (age 15–49 years and, in most cases, ever married) was identified for survey participation. Detailed sampling plans are available from survey final reports for each nation and year [16]–[28]. For each sampled household, a household questionnaire was also administered. Weighting was conducted to account for the multistage sampling design. To understand girl child marriage for the nations and time points selected, we limited our sample to ever married 20–24 year old women for each survey included. This age range allows for measurement of early relative to delayed marriage without bias towards over-inclusion of those marrying as minors, and for a sample young enough to reflect recent practices relative to the survey years. An ever married sample was used because the majority of the surveys only sampled ever married women. Sample sizes for nation stratified analyses were, for Bangladesh, N = 8,002 (1996–1997 n = 1,716; 1999–2000 n = 1,910; 2004 n = 2,202; 2007 n = 2,174;), for India, N = 48,004 (1992–1993 n = 17,218; 1998–1999 n = 15,973; 2005–2006 n = 14,813), for Nepal, N = 6,774 (1996 n = 1,629; 2001 n = 1,651; 2006 n = 1,679; 2011 n = 1,715), and for Pakistan, N = 2,624 (1990–1991 n = 1,064; 2006–2007 n = 1,560) [16]–[28]. The 1993–1994 Bangladesh DHS was excluded as data collected did not include all measures required for the present analyses.

### Measures

Our outcome of focus was *girl child marriage* which was operationalized as a categorical variable based on age at marriage (<14 years, 14–15 years, 16–17 years and ≥18 years), to allow consideration of factors associated with marriage at younger and older adolescent minor ages. This variable was calculated as the difference between the age of the participant and the age at which she began living with her first husband. Our primary exposure variable was *education*, which was based on a single item assessing the highest education level they had attended- no education, primary, secondary, or higher. The categories are not indicative of completing the level of education; education completion is not assessed in the DHS. However, a second DHS item (included in post-hoc analyses) assessed the number of years of education completed at the highest level of education attended, among those reporting receipt of education.

To ensure observed associations between our primary exposure and outcome variables were not an artifact of related social or gender inequity indicators, we included the following covariates: urban/rural residence, state/region of residence, wealth quintile, and gaps in spousal age and education. Wealth quintiles were calculated at the household level using principal components analysis based on assessments of housing characteristics and household assets as observed by the interviewer [31]. Spousal age gap was based on the difference in age between current husband and respondent, which was then dichotomized as <10 years or 10+ years difference based on previous research indicating the association between this spousal age gap and girl child marriage in India [32]. Spousal education gap was calculated as the difference between husbands and wives regarding the number of years of education received.

### Statistical analysis

To assess the effect of education level on age at marriage, multinomial logistic regression models stratified by country were constructed, including all variables previously described as well as survey year. Multinomial regression was chosen instead of ordinal regression as we did not necessarily expect the increases in odds to be linear across the age at marriage categories. Additionally, the interaction between survey year and education level was included in all models, as it was significant at the <0.05 level in India, Nepal and Pakistan. The interaction term was included for Bangladesh for comparability across models.

A post-hoc exploratory analysis was also conducted in which the multinomial logistic regression models previously described were run that included an indicator of both level of education and years of education. In this exploratory model, based on observed effects of secondary education, education was categorized as no education, any primary education, 0–1, 2, 3, 4 or ≥5 completed years of secondary education, and any higher education. All analyses were adjusted for complex sample design and individual weights. Analyses were conducted using SAS v. 9.2 and Stata 12.

## Results

### Sample Characteristics

**Figure 1:**
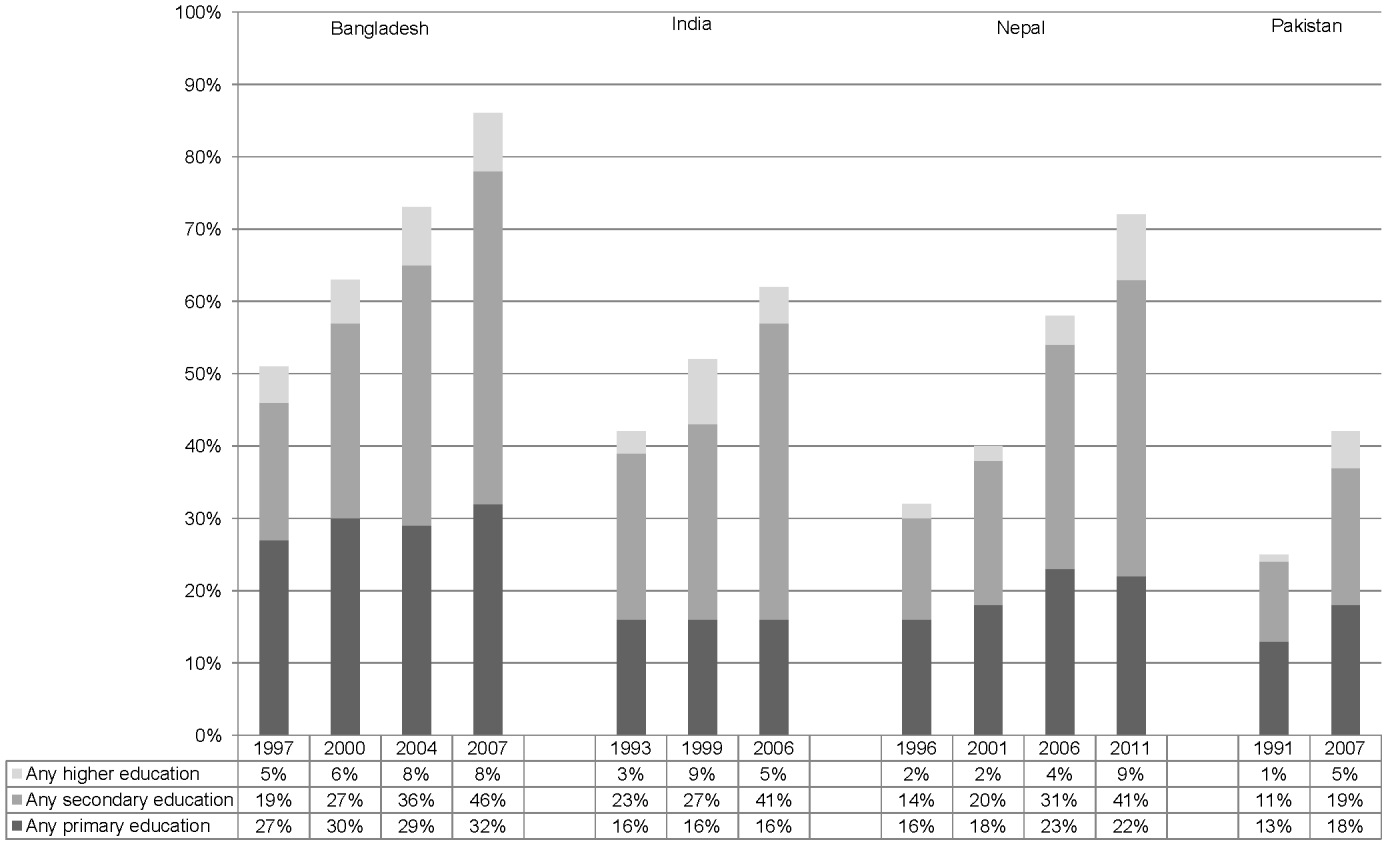
Education levels of ever married 20–24 year old women in Bangladesh, India, Nepal, and Pakistan, 1991–2011.

Most recent data across the nations of focus indicate that 50–77% of this ever married sample was married prior to age 18 years, with those in Bangladesh most likely and those in Pakistan least likely to be married as minors. (See Table 1.) No receipt of education was reported by 15–58% of most recent survey respondents, with women from Bangladesh being most likely and women from Pakistan least likely to report any education. Education did however increase for all nations over the survey timeframe, most dramatically for Bangladesh. (See Figure 1 and Tables S1–S4.) Most recent data indicate that education levels advantage husbands over wives for all countries but Bangladesh, where wives are advantaged. Large (10+ years) spousal age gaps advantaging husbands is also most common in Bangladesh, where 41% of the sample report this phenomenon relative to 9 to 19% of those in other nations. The majority of participants across all nations resided in rural areas.

**Table 1:** Demographic profiled of ever-married women aged 20–24 years for most recent Demographic Health Survey data from Bangladesh, India, Nepal, and Pakistan.

	Bangladesh 2007	India 2005–06	Nepal 2011	Pakistan 2006–07
	N = 2174	N = 14813	N = 1715	N = 1560
	Weighted % (95% CI)	Weighted % (95% CI)	Weighted % (95% CI)	Weighted % (95% CI)
Age				
20	22% (20%–24%)	20% (19%–21%)	15% (13%–17%)	23% (20%–25%)
21	20% (18%–22%)	17% (17%–18%)	20% (18%–22%)	14% (12%–16%)
22	21% (18%–23%)	21% (21%–22%)	23% (21%–25%)	24% (22%–26%)
23	20% (18%–22%)	21% (20%–21%)	19% (17%–22%)	17% (15%–18%)
24	18% (17%–20%)	20% (20%–21%)	22% (20%–25%)	23% (20%–25%)
Education level				
None	15% (13%–18%)	39% (38%–41%)	28% (24%–33%)	58% (55%–61%)
Any primary	32% (29%–34%)	16% (15%–16%)	22% (19%–25%)	18% (16%–20%)
Any secondary	46% (43%–48%)	41% (39%–42%)	41% (37%–45%)	19% (17%–21%)
Any higher	8% (6%–9%)	5% (4%–5%)	9% (7%–11%)	5% (4%–6%)
Rural residence	77% (75%–79%)	73% (72%–74%)	89% (88%–90%)	70% (68%–72%)
Wealth quintile				
Poorest	18% (16%–20%)	20% (19%–21%)	16% (13%–19%)	20% (17%–22%)
Poorer	19% (17%–21%)	22% (21%–23%)	19% (16%–23%)	23% (21%–25%)
Middle	21% (19%–23%)	22% (21%–23%)	22% (19%–25%)	20% (18%–22%)
Richer	22% (20%–25%)	21% (20%–22%)	25% (21%–29%)	19% (17%–21%)
Richest	20% (17%–22%)	15% (14%–16%)	17% (14%–21%)	18% (16%–20%)
Spousal Age gap†	41% (38%–43%)	14% (13%–15%)	9% (7%–11%)	19% (17%–21%)
Education gap§	−0.7 (−3.4, 1.4)	0.5 (−0.8, 4.4)	0.4 (−0.8, 3.1)	2.0 (−0.5, 6.3)
Age at Marriage				
<14	22% (19%–24%)	8% (8%–9%)	5% (4%–6%)	5% (4%–6%)
14–15	32% (29%–34%)	22% (21%–23%)	20% (17%–23%)	18% (17%–20%)
16–17	24% (22%–26%)	29% (28%–30%)	28% (25%–31%)	26% (24%–29%)
18 and older	23% (21%–25%)	41% (40%–42%)	47% (44%–51%)	50% (47%–52%)

† ≥10 year age gap between husband and wife.

§ Years of completed education of wife subtracted from years of completed education of husband; Median and IQR.

### Multinomial analyses to assess associations between education and girl child marriage

In Bangladesh and Pakistan, primary education was not protective against early marriage, and in Nepal, primary education was only protective for very young marriages <14 years (adjusted odds ratio [AOR] = 0.42, 95% CI = 0.24, 0.76). [See Table 2.] Secondary education in Bangladesh and Nepal was protective across minor age at marriage categories (Bangladesh <14 years AOR = 0.10, 95% CI = 0.06, 0.14; 14–15 years AOR = 0.25, 95% CI = 0.17, 0.36; 16–17 years AOR = 0.64, 95% CI = 0.43, 0.96; Nepal <14 years AOR = 0.21, 95% CI = 0.11, 0.41; 14–15 years AOR = 0.25, 95% CI = 0.18, 0.35; 16–17 years AOR = 0.57, 95% CI = 0.41, 0.77 ), but only protective against marriage of younger adolescents and girls in Pakistan (<14 years AOR = 0.19, 95% CI = 0.06, 0.64; 14–15 years AOR = 0.23, 95% CI = 0.10, 0.54). In India, both primary and secondary education were protective across all minor age at marriage categorizations (e.g., India primary education for 16–17 year olds AOR = 0.71, 95% CI = 0.63, 0.80 and secondary education for 16–17 year olds AOR = 0.48, 95% CI = 0.43, 0.54).

**Table 2:** Multinomial model assessing odds of marrying at ages <14, 14–15, 16–17, compared to marrying at 18 or older in Bangladesh, India, Nepal and Pakistan.

	<14 yrs	14–15 yrs	16–17 yrs
	AOR (95% CI)	AOR (95% CI)	AOR (95% CI)
**BANGLADESH**			
**Education level**			
No education	[REF]	[REF]	[REF]
Any primary education	0.70 (0.48,1.03)	0.78 (0.53,1.15)	1.04 (0.67,1.61)
Any secondary education	0.10 (0.06,0.14)	0.25 (0.17,0.36)	0.64 (0.43,0.96)
Any higher education†	0.02 (0.01,0.06)	0.02 (0.01,0.06)	0.22 (0.12,0.40)
**Time (survey year)**	0.97 (0.92,1.03)	1.04 (0.99,1.10)	1.04 (0.98,1.10)
**Education gap** §	0.97 (0.95,0.99)	0.97 (0.94,0.99)	0.99 (0.96,1.01)
**Type of residence**			
Urban	[REF]	[REF]	[REF]
Rural	1.21. (0.98,1.50)	1.20 (0.98,1.47)	1.10 (0.90,1.34)
**Wealth quintile**			
Richest	[REF]	[REF]	[REF]
Richer	1.37 (1.06,1.77)	1.21 (0.94,1.54)	1.19 (0.93,1.51)
Middle	1.37 (1.04,1.82)	1.28 (0.98,1.68)	1.33 (1.02,1.74)
Poorer	2.04 (1.52,2.75)	1.72 (1.26,2.33)	1.17 (0.86,1.60)
Poorest	2.31 (1.66,3.20)	1.97 (1.40,2.76)	1.51 (1.07,2.13)
**Age gap**			
<10 years	[REF]	[REF]	[REF]
≥10 years	2.91 (2.42,3.49)	2.30 (1.94,2.73)	1.62 (1.37,1.93)
**State of residence**			
Rajashani	[REF]	[REF]	[REF]
Barisal	0.45 (0.34,0.60)	0.62 (0.47,0.83)	0.73 (0.56,0.95)
Chittagong	0.17 (0.14,0.23)	0.37 (0.29,0.47)	0.56 (0.44,0.71)
Dhaka	0.55 (0.43,0.70)	0.67 (0.52,0.86)	0.77 (0.61,0.97)
Khulna	1.04 (0.78,1.39)	1.26 (0.92,1.73)	1.19 (0.90,1.57)
**INDIA**			
**Education level**			
No education	[REF]	[REF]	[REF]
Any primary education	0.34 (0.28,0.42)	0.52 (0.46,0.60)	0.71 (0.63,0.80)
Any secondary education	0.05 (0.04,0.07)	0.20 (0.18,0.23)	0.48 (0.43,0.54)
Any higher education†	0.01 (0.00,0.07)	0.03 (0.01,0.07)	0.16 (0.12,0.22)
**Time (survey year)**	0.98 (0.97,1.00)	1.00 (0.99,1.01)	1.01 (1.00,1.02)
**Education gap** §	0.96 (0.95,0.97)	0.99 (0.98,1.00)	1.00 (0.99,1.01)
**Type of residence**			
Urban	[REF]	[REF]	[REF]
Rural	1.29 (1.11,1.50)	1.15 (1.04,1.27)	0.99 (0.91,1.07)
**Wealth quintile**			
Richest	[REF]	[REF]	[REF]
Richer	1.47 (1.17,1.85)	1.71 (1.49, 1.95)	1.33 (1.21,1.46)
Middle	2.47 (1.94,3.15)	2.70 (2.34,3.12)	1.74 (1.56,1.95)
Poorer	3.24 (2.48,4.22)	3.32 (2.84,3.88)	2.04 (1.81,2.30)
Poorest	3.50 (2.67,4.58)	3.46 (2.92,4.11)	2.15 (1.87,2.46)
**Age gap**			
<10 years	[REF]	[REF]	[REF]
≥10 years	2.81 (2.49,3.17)	1.87 (1.70,2.05)	1.29 (1.18,1.41)
**Region of residence**			
West	[REF]	[REF]	[REF]
Central	0.84 (0.70,1.01)	0.97 (0.85,1.10)	1.07 (0.96,1.18)
East	0.68 (0.56,0.82)	0.79 (0.68,0.92)	0.96 (0.86,1.07)
North	0.57 (0.47,0.69)	0.67 (0.59,0.77)	0.84 (0.76,0.94)
Northeast	0.47 (0.36,0.62)	0.65 (0.54,0.79)	0.76 (0.66,0.86)
South	1.06 (0.89,1.28)	0.91 (0.80,1.05)	0.92 (0.84,1.02)
**NEPAL**			
**Education level**			
No education	[REF]	[REF]	[REF]
Any primary education	0.42 (0.24,0.76)	0.73 (0.52,1.01)	0.97 (0.74,1.27)
Any secondary education	0.21 (0.11,0.41)	0.25 (0.18,0.35)	0.57 (0.41,0.77)
Any higher education†	0.05 (0.00,0.62)	0.02 (0.00,0.14)	0.04 (0.04,0.23)
**Time (survey year)**	0.94 (0.91,0.98)	0.99 (0.97,1.01)	0.99 (0.99,1.03)
**Education gap** §	0.99 (0.95,1.03)	1.00 (0.98,1.03)	0.97 (0.97,1.02)
**Type of residence**			
Urban	[REF]	[REF]	[REF]
Rural	0.58 (0.38,0.90)	0.77 (0.59,1.00)	0.83 (0.66,1.05)
**Wealth quintile**			
Richest	[REF]	[REF]	[REF]
Richer	1.81 (1.05,3.16)	1.11 (0.85,1.42)	0.95 (0.95,1.54)
Middle	1.97 (1.08,3.60)	1.57 (1.18,2.09)	1.51. (1.15,1.97)
Poorer	2.15 (1.21,3.82)	1.55 (1.18,2.04)	1.48 (1.14,1.92)
Poorest	1.85 (1.03,3.34)	1.35 (1.00,1.81)	1.46 (1.11,1.92)
**Age gap**			
<10 years	[REF]	[REF]	[REF]
≥10 years	1.46 (0.95,2.24)	1.35 (1.04,1.76)	1.08 (0.85,1.38)
**State of residence**			
Western	[REF]	[REF]	[REF]
Central	1.27 (0.84,1.92)	1.30 (1.03,1.65)	0.74 (0.74,1.15)
Eastern	1.11 (0.73,1.67)	0.78 (0.60,1.01)	0.64 (0.51,0.81)
Farwestern	1.37 (0.79,2.36)	1.04 (0.67,1.62)	0.50 (0.50,1.34)
Midwestern	1.17 (0.74,1.85)	1.14 (0.89,1.46)	1.03 (0.82,1.30)
**PAKISTAN**			
**Education level**			
No education	[REF]	[REF]	[REF]
Any primary education	0.68 (0.34,1.33)	0.72 (0.38,1.35)	0.94 (0.48,1.85)
Any secondary education	0.19 (0.06,0.64)	0.23 (0.10,0.54)	0.58 (0.30,1.11)
Any higher education†	0.00 (0.00,0.00)	0.00 (0.00,0.00)	0.00 (0.00,0.00)
**Time (survey year)**	0.96 (0.94,0.99)	0.99 (0.97,1.01)	1.02 (1.00,1.04)
**Education gap** §	0.97 (0.94,1.01)	1.00 (0.97,1.04)	1.00 (0.98,1.03)
**Type of residence**			
Urban	[REF]	[REF]	[REF]
Rural	0.80 (0.51,1.25)	0.85 (0.60,1.20)	0.98 (0.74,1.31)
**Wealth quintile**			
Richest	[REF]	[REF]	[REF]
Richer	1.93 (1.08,3.34)	1.18 (0.75,1.85)	1.56 (1.06,2.30)
Middle	2.67 (1.43,5.00)	1.98 (1.16,3.38)	1.92 (1.20,3.05)
Poorer	2.42 (1.22,4.79)	2.76 (1.64,4.62)	1.93 (1.19,3.15)
Poorest	3.62 (1.71,7.65)	3.77 (2.13,6.70)	2.53 (1.51,4.25)
**Age gap**			
<10 years	[REF]	[REF]	[REF]
≥10 years	2.67 (1.75,4.07)	2.24 (1.64,3.07)	2.24 (1.64,3.07)
**State of residence**			
Sindh	[REF]	[REF]	[REF]
Balochistan	0.59 (0.35,1.02)	0.49 (0.33,0.74)	0.65 (0.47,0.89)
NWFP	0.77 (0.46,1.30)	0.88 (0.63,1.25)	0.83 (0.58,1.17)
Punjab	0.30 (0.19,0.48)	0.48 (0.36,0.64)	0.60 (0.46,0.79)

Adjusted for education level by survey year interaction. Interactions terms were significant (p<.05) for India, Nepal, Pakistan.

† Higher education estimates may be inaccurate due to small cell sizes.

§ Years of completed education of wife subtracted from years of completed education of husband.

**Figure 2:**
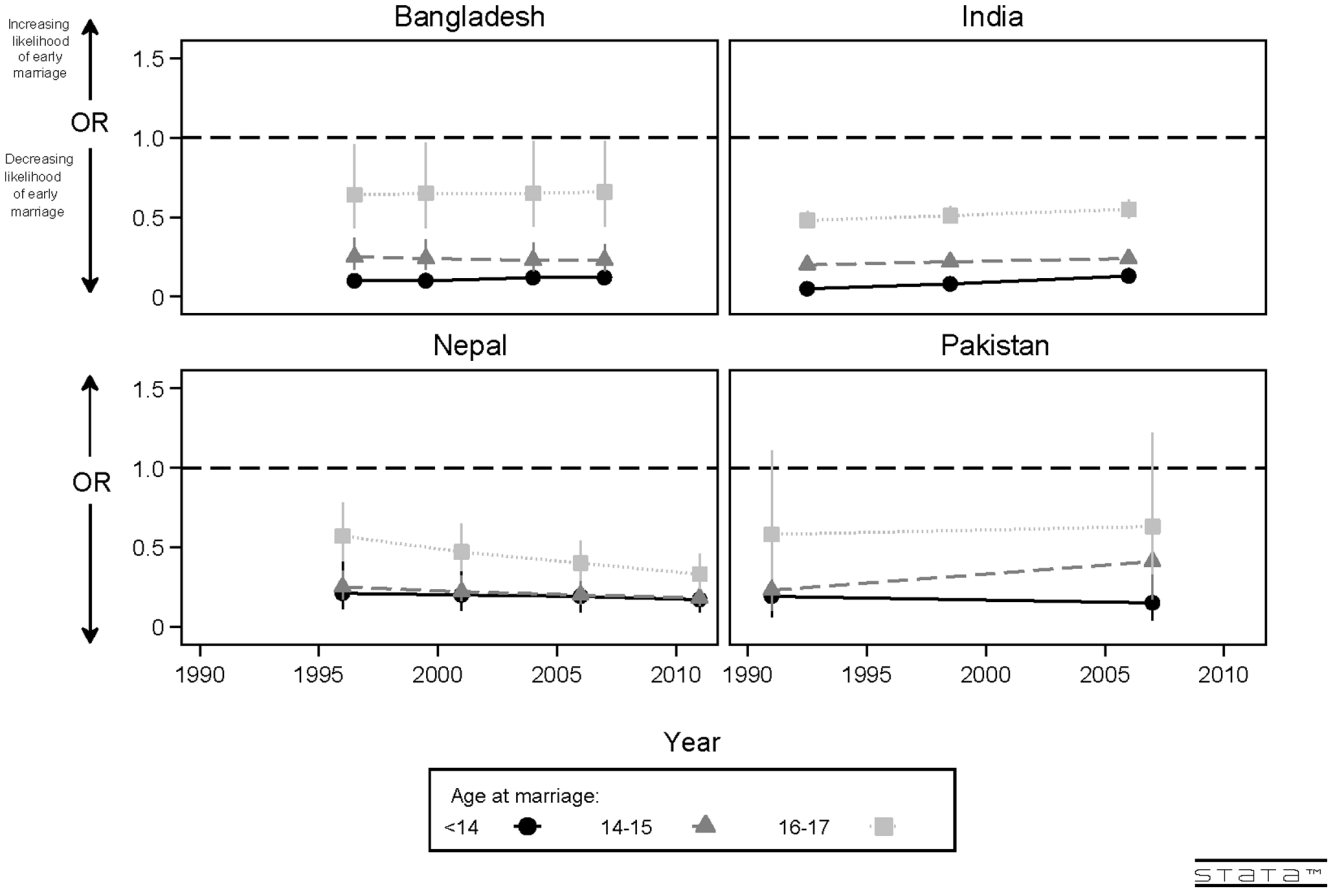
Adjusted odds ratios showing associations between secondary education and girl child marriage, 1991 to 2011.

Due to stronger findings for secondary rather than primary education, and based on the significant interactions between survey year and education for all countries with the exception of Bangladesh, adjusted odds ratios documenting the associations between secondary education and girl child marriage were graphed over time to determine whether the associations are time-stable or altering. (See Figure 2.) Graphs show that, while there were increasing and decreasing trends in the odds ratios over time, secondary education remains protective for girl child marriage across time in all countries. In Bangladesh, secondary education remains significantly protective of girl child marriage for all minor age at marriage categorizations, with very little change in the magnitude of the association. In India, secondary education, while significantly protective for girl child marriage across time and minor age at marriage categorizations, shifts upwards over time (towards an OR of 1.0), suggesting that, while still beneficial, the magnitude of the impact is decreasing slightly over time. Conversely, in Nepal, secondary education has become more protective for marriage at 16–17 years over time. In Pakistan, numbers were small, but there appeared to be some decrease in the impact of secondary education over time on marriage at 14–15 years, and some increase in the impact over time on marriage at <14.

To further clarify the secondary education findings, particularly for older adolescents (i.e., 16–17 year olds) where education effects are diminished, a post-hoc multivariate model was constructed in which the education variable included number of years of secondary education. For Bangladesh, no significant associations were seen unless three or more years of secondary education were provided [3 years of secondary education AOR = 0.51, 95% CI = 0.28, 0.93; 4 years = 0.56, 95% CI = 0.34, 0.58; 5+ years AOR = 0.30, 95% CI = 0.16, 0.58]. (See Table S5.) For Nepal, no significant associations were seen unless four or more years of secondary education were provided [4 years of secondary education AOR = 0.37, 95% CI = 0.22, 0.61; 5+ years AOR = 0.28, 95% CI = 0.17, 0.45]. For India, associations were seen for each year of secondary education received [0–1 year of secondary education AOR = 0.66, 95% CI = 0.56, 0.77; 2 years AOR = 0.50, 95% CI = 0.40, 0.62; 3 years AOR = 0.39, 95% CI = 0.32, 0.48), 4 years = 0.56, 95% CI = 0.34, 0.58; 5+ years AOR = 0.30, 95% CI = 0.16, 0.58]. Reliable estimates could not be obtained for Pakistan due to small numbers.

## Discussion

Study findings document varying effects of education on girl child marriage across South Asia by nation and age of girls at marriage. Primary education was protective against early marriage in India, but not in Bangladesh, Nepal or Pakistan. These findings indicate a likely reduction in the protective effects of this most basic level of education, as previous research suggested that primary education was protective against marriage for younger adolescents in the prior decade for both Bangladesh and Nepal [3]. Consistent with this prior work, however, current findings document that secondary education is protective against early marriage for both younger girls and older adolescents in Bangladesh, India and Nepal, and for younger adolescents in Pakistan, with current time trend analyses further indicating stability or improvement of these effects over the past 20 years. Overall, these findings suggest that emphasis on girl participation in secondary education, and not just primary education, is required to reduce further girl child marriage in South Asia. While there are variations in levels of girl participation and gender parity in education across these South Asian countries of focus [13], the importance of secondary education appears meaningful for all of these nations. Given recent international commitments of $1.6 billion to the UN to increase and improve universal education [33] and the recently announced intensification of the efforts of US State Department to reduce child marriage via increased girl education, the current findings offer important guidance. Resources for girls’ secondary education should be prioritized in South Asia, where universal education has not been achieved and where education gender gaps remain a concern for India and Pakistan [10], [12], [34].

Importantly, while current findings support the utility of secondary education for girls regarding reduction of girl child marriage, it also indicates that these effects, while significant, are modest. Marginal effects based on study findings were applied to population estimates for each nation [19], [22], [26], [28], [35] and indicate that if all girls received secondary education, there would be an expected reduction of 446,567 of an expected 6.9 million girl child marriages, or a 6.5% reduction of early marriages in the region. (See Table S6 for details.) The modest size of this observed effect contrasts sharply with the expectations of current international efforts to reduce this harmful practice and to, thus, reduce maternal and child morbidity and mortality [6]–[8]. The potentially inadequate benefits of secondary education alone are likely in part attributable to their lesser protective effects for older relative to younger adolescents, particularly when fewer years of secondary education are received. Older adolescents are the majority of those marrying as minors, and proportions of girls marrying in older adolescence (16–17 years) have not diminished in the region in the past 20 years [11]. These findings demonstrate that while education is necessary, it alone will not be sufficient to create the desired impact of eliminating girl child marriage in South Asia. Results of other research on gender equality and women’s health, not specific to early marriage, similarly find that education alone is likely insufficient to achieve major improvements in health outcomes for women outside of “settings which are already less patriarchal, where women have access to services, options and opportunities, and where market and social conditions favor positive returns [4].” Such findings are consistent with models of intervention thought to effectively prevent child marriage, which focus on gender equity ideologies, life skills training, and improved economic opportunities for girls, often in conjunction with improved education [5], [6], [13]. The importance of focus on gender and economic empowerment approaches is underscored by current analyses documenting that even after controlling for education, issues of poverty (social inequity) and spousal age gap (gender inequity) remain associated with girl child marriage. However, simultaneously, cultural issues that reinforce the practice must be addressed as well, and likely require affecting decision-makers for girls, such as parents or community (e.g., religious or tribal) leaders, and not just the girls themselves.

The current analyses have several limitations that should be considered. Only Nepal analyses included data from 2011; for Bangladesh, India and Pakistan, most recent data available were from 2005–2007. However, given that findings from this work indicate that associations between education and girl child marriage are not dramatically altering over time, findings using 2005–2007 data are unlikely very different from those that may be observed with more recent data. Causality cannot be assumed from current findings, as analyses are cross-sectional; longitudinal research is required to provide more insight into this issue. An additional limitation is the restriction of the sample to ever married women, as the majority of available survey data was only available from this group. This approach, however, would likely yield more rather than less conservative estimates, as unmarried women are more likely to have higher education than those married at younger ages in these national contexts [3], [19], [22], [26], [28]. Higher education was unable to receive adequate focus in this study because, though these data were available and included, small numbers limited accuracy of estimates for that group. Quality of education was also not assessed via these measures. DHS data are susceptible to social desirability and recall bias, though the latter should be minimized due to the relatively recent nature of the events being analyzed. Social desirability, however, may have varied over time regarding age at marriage and education, as norms likely shifted on these factors across years of study.

### Conclusion

Secondary education is associated with reduced risk for girl child marriage in Bangladesh, India, Nepal and Pakistan, but these effects, while significant, are not large and are lesser for older adolescents relative to younger girls. Such findings indicate that universal high quality secondary education may be gender-transformative and is likely necessary to achieve reductions in early marriage among girls in South Asia, but by itself it will be insufficient to achieve the global goal of elimination of girl child marriage by 2030, the current goal of major international stakeholders [8]. Further research is needed to guide additional intervention approaches to eliminate girl child marriage in the region. Programs and an environment supporting girls’ and women’s social and economic empowerment, in conjunction with improved girl education, will likely be required to eliminate girl child marriage in South Asia.

## Supporting Information

Table S1 **Sample characteristics of ever-married women aged 20–24 years in Bangladesh, 1997, 2000, 2004, and 2007.**
(DOCX)

Table S2 **Sample characteristics of ever-married women aged 20–24 years in India, 1993, 1999 and 2006.**
(DOCX)

Table S3 **Sample characteristics of ever-married women aged 20–24 years in Nepal, 1996, 2001, 2006, and 2011.**
(DOCX)

Table S4 **Sample characteristics of ever-married women aged 20–24 years in Pakistan, 1991 and 2007.**
(DOCX)

Table S5 **Odds of girl child marriage in Bangladesh, India, Nepal and Pakistan by expanded education level.**
(DOCX)

Table S6 **Annual reductions in girl child marriages attributable to universal access to secondary education.**
(DOCX)
