# Supplementary material for: Cross-Sectional Time Series Analysis of Associations between Education and Girl Child Marriage in Bangladesh, India, Nepal and Pakistan, 1991-2011
Source: PLoS One. 2014 Sep 9;9(9):e106210. doi: 10.1371/journal.pone.0106210 (PMC4159189; doi:10.1371/journal.pone.0106210)
Supplement: Table S1 — Sample characteristics of ever-married women aged 20–24 years in Bangladesh, 1997, 2000, 2004, and 2007. (DOCX) [file pone.0106210.s001.docx]

**Appendix Table S1. Sample characteristics of ever-married women aged 20-24 years in Bangladesh, 1997, 2000, 2004, and 2007.**

|  | 1997 | 2000 | 2004 | 2007 |
| --- | --- | --- | --- | --- |
|  | N=1715 | N=1910 | N=2202 | N=2174 |
|  | Weighted percentage (95% CI) | Weighted percentage (95% CI) | Weighted percentage (95% CI) | Weighted percentage (95% CI) |
| Age at marriage |  |  |  |  |
| <14 | 42% (40%-45%) | 31% (28%-33%) | 29% (26%-31%) | 22% (19%-24%) |
| 14-15 | 25% (23%-28%) | 30% (28%-32%) | 30% (28%-32%) | 32% (29%-34%) |
| 16-17 | 15% (13%-17%) | 19% (18%-21%) | 22% (20%-24%) | 24% (22%-26%) |
| ≥18 | 17% (15%-19%) | 20% (18%-22%) | 19% (17%-21%) | 23% (21%-25%) |
| Age at Interview |  |  |  |  |
| 20 | 20% (18%-22%) | 21% (19%-23%) | 21% (19%-24%) | 22% (20%-24%) |
| 21 | 20% (18%-23%) | 20% (18%-22%) | 20% (17%-22%) | 20% (18%-22%) |
| 22 | 20% (18%-22%) | 21% (18%-23%) | 22% (20%-24%) | 21% (18%-23%) |
| 23 | 19% (18%-21%) | 20% (18%-22%) | 17% (16%-19%) | 20% (18%-22%) |
| 24 | 21% (19%-23%) | 18% (17%-20%) | 19% (17%-21%) | 18% (17%-20%) |
| Education level |  |  |  |  |
| None | 50% (47%-52%) | 38% (35%-40%) | 27% (24%-29%) | 15% (13%-18%) |
| Any primary education | 27% (25%-29%) | 30% (27%-32%) | 29% (27%-31%) | 32% (29%-34%) |
| Any secondary education | 19% (17%-21%) | 27% (24%-29%) | 36% (34%-39%) | 46% (43%-48%) |
| Any higher education | 5% (4%-6%) | 6% (5%-8%) | 8% (7%-10%) | 8% (6%-9%) |
| Rural residence | 89% (88%-91%) | 81% (79%-83%) | 77% (75%-79%) | 77% (75%-79%) |
| Wealth quintile |  |  |  |  |
| Poorest | 19% (17%-22%) | 22% (20%-24%) | 21% (19%-23%) | 18% (16%-20%) |
| Poorer | 22% (20%-24%) | 20% (18%-22%) | 17% (15%-19%) | 19% (17%-21%) |
| Middle | 20% (18%-22%) | 19% (17%-22%) | 19% (17%-22%) | 21% (19%-23%) |
| Richer | 19% (17%-21%) | 19% (17%-21%) | 20% (18%-22%) | 22% (20%-25%) |
| Richest | 20% (17%-22%) | 20% (17%-22%) | 23% (20%-25%) | 20% (17%-22%) |
| Age gap† | 40% (37%-43%) | 43% (41%-46%) | 44% (42%-46%) | 41% (38%-43%) |
| Education gap *§* | -0.3 (-0.8-1.9) | -0.3 (-1.0-1.8) | -0.4 (-2.3-1.6) | -0.7 (-3.4-1.4) |

*†≥10 year age gap between husband and wife ± Median and IQR §Years of completed education of wife subtracted from years of completed education of husband; Median and IQR*
