# Supplementary material for: Cross-Sectional Time Series Analysis of Associations between Education and Girl Child Marriage in Bangladesh, India, Nepal and Pakistan, 1991-2011
Source: PLoS One. 2014 Sep 9;9(9):e106210. doi: 10.1371/journal.pone.0106210 (PMC4159189; doi:10.1371/journal.pone.0106210)
Supplement: Table S2 — Sample characteristics of ever-married women aged 20–24 years in India, 1993, 1999 and 2006. (DOCX) [file pone.0106210.s002.docx]

**Appendix Table S2. Sample characteristics of ever-married women aged 20-24 years in India, 1993, 1999 and 2006.**

|  | 1993 | | 1999 | | 2006 |
| --- | --- | --- | --- | --- | --- |
|  | N=17214 | | N=15973 | | N=14813 |
|  | Weighted percentage (95% CI) | | Weighted percentage (95% CI) | | Weighted percentage (95% CI) |
| Age at marriage |  | |  | |  |
| <14 | 12% (11%-12%) | | 10% (10%-11) | | 8% (8%-9%) |
| 14-15 | 24% (23%-25%) | | 24% (23%-25%) | | 22% (21%-23%) |
| 16-17 | 26% (25%-27%) | | 25% (24%-26%) | | 29% (28%-30%) |
| ≥18 | 38% (37%-39%) | | 41% (40%-42%) | | 41% (40%-42%) |
| Age at Interview |  | |  | |  |
| 20 | 23% (22%-23%) | | 21% (20%-22%) | | 20% (19%-21%) |
| 21 | 18% (17%-18%) | | 18% (17%-19%) | | 17% (17%-18%) |
| 22 | 21% (20%-21%) | | 21% (20%-22%) | | 21% (21%-22%) |
| 23 | 19% (18%-19%) | | 20% (19%-21%) | | 21% (20%-21%) |
| 24 | 20% (20%-21%) | | 20% (19%-21%) | | 20% (20%-21%) |
| Education level |  | |  | |  |
| None | 58% (57%-59%) | | 47% (46%-49%) | | 39% (38%-41%) |
| Any primary education | 16% (15%-17%) | | 16% (15%-17%) | | 16% (15%-16%) |
| Any secondary education | 23% (22%-24%) | | 27% (27%-28%) | | 41% (39%-42%) |
| Any higher education | 3% (2%-3%) | | 9% (8%-10%) | | 5% (4%-5%) |
| Rural residence | 76% (75%-78%) | | 77% (75%-79%) | | 73% (72%-74%) |
| Wealth quintile |  |  |  |  |  |
| Poorest | 19% (18%-20%) | | 20% (19%-21%) | | 20% (19%-21%) |
| Poorer | 22% (21%-22%) | | 21% (20%-22%) | | 22% (21%-23%) |
| Middle | 22% (21%-23%) | | 21% (20%-22%) | | 22% (21%-23%) |
| Richer | 21% (20%-22%) | | 21% (20%-22%) | | 21% (20%-22%) |
| Richest | 17% (16%-18%) | | 17% (16%-18%) | | 15% (14%-16%) |
| Age gap† | 15% (14%-16%) | | 17% (16%-17%) | | 14% (13%-15%) |
| Education gap *§* | 0.6 (-0.6-4.8) | | 1.0 (-0.7-4.7) | | 0.5 (-0.8-4.4) |

*†≥10 year age gap between husband and wife ± Median and IQR §Years of completed education of wife subtracted from years of completed education of husband; Median and IQR*
