# Supplementary material for: Cross-Sectional Time Series Analysis of Associations between Education and Girl Child Marriage in Bangladesh, India, Nepal and Pakistan, 1991-2011
Source: PLoS One. 2014 Sep 9;9(9):e106210. doi: 10.1371/journal.pone.0106210 (PMC4159189; doi:10.1371/journal.pone.0106210)
Supplement: Table S3 — Sample characteristics of ever-married women aged 20–24 years in Nepal, 1996, 2001, 2006, and 2011. (DOCX) [file pone.0106210.s003.docx]

**Appendix Table S3. Sample characteristics of ever-married women aged 20-24 years in Nepal, 1996, 2001, 2006, and 2011.**

|  | 1996 | 2001 | 2006 | 2011 |
| --- | --- | --- | --- | --- |
|  | N=1629 | N=1651 | N=1679 | N=1715 |
|  | Weighted percentage (95% CI) | Weighted percentage (95% CI) | Weighted percentage (95% CI) | Weighted percentage (95% CI) |
| Age at marriage |  |  |  |  |
| <14 | 10% (8%-12%) | 4% (3%-5%) | 5% (3%-6%) | 5% (4%-6%) |
| 14-15 | 30% (27%-33%) | 30% (27%-32%) | 23% (20%-25%) | 20% (17%-23%) |
| 16-17 | 31% (28%-33%) | 34% (31%-36%) | 35% (31%-39%) | 28% (25%-31%) |
| ≥18 | 29% (27%-32%) | 32% (30%-35%) | 37% (33%-42%) | 47% (44%-51%) |
| Age at Interview |  |  |  |  |
| 20 | 20% (18%-22%) | 19% (17%-21%) | 16% (13%-18%) | 15% (13%-17%) |
| 21 | 17% (16%-19%) | 20% (18%-22%) | 19% (17%-22%) | 20% (18%-22%) |
| 22 | 22% (20%-24%) | 23% (21%-25%) | 23% (21%-26%) | 23% (21%-25%) |
| 23 | 18% (16%-20%) | 19% (17%-21%) | 18% (16%-21%) | 19% (17%-22%) |
| 24 | 23% (21%-25%) | 20% (18%-22%) | 23% (20%-25%) | 22% (20%-25%) |
| Education level |  |  |  |  |
| None | 68% (65%-72%) | 59% (55%-63%) | 41% (38%-45%) | 28% (24%-33%) |
| Any primary education | 16% (14%-18%) | 18% (16%-20%) | 23% (21%-26%) | 22% (19%-25%) |
| Any secondary education | 14% (11%-17%) | 20% (17%-23%) | 31% (28%-34%) | 41% (37%-45%) |
| Any higher education | 2% (1%-3%) | 2% (2%-3%) | 4% (3%-5%) | 9% (7%-11%) |
| Rural residence | 92% (89%-95%) | 90% (88%-93%) | 85% (83%-87%) | 89% (88%-90%) |
| Wealth quintile |  |  |  |  |
| Poorest | 19% (16%-22%) | 20% (18%-23%) | 18% (15%-21%) | 16% (13%-19%) |
| Poorer | 20% (18%-22%) | 20% (18%-23%) | 20% (17%-23%) | 19% (16%-23%) |
| Middle | 21% (19%-23%) | 20% (17%-22%) | 20% (17%-23%) | 22% (19%-25%) |
| Richer | 21% (19%-24%) | 20% (18%-23%) | 21% (18%-25%) | 25% (21%-29%) |
| Richest | 18% (14%-21%) | 20% (16%-23%) | 20% (17%-24%) | 17% (14%-21%) |
| Age gap† | 9% (8%-11%) | 7% (6%-8%) | 9% (7%-11%) | 41% (38%-45%) |
| Education gap *§* | 1.9 (-0.4-5.4) | 2.0 (-0.4-5.1) | 1.5 (-0.4-4.5) | 0.4 (-0.8-3.1) |

*†≥10 year age gap between husband and wife ± Median and IQR §Years of completed education of wife subtracted from years of completed education of husband; Median and IQR*
