# Supplementary material for: Cross-Sectional Time Series Analysis of Associations between Education and Girl Child Marriage in Bangladesh, India, Nepal and Pakistan, 1991-2011
Source: PLoS One. 2014 Sep 9;9(9):e106210. doi: 10.1371/journal.pone.0106210 (PMC4159189; doi:10.1371/journal.pone.0106210)
Supplement: Table S4 — Sample characteristics of ever-married women aged 20–24 years in Pakistan, 1991 and 2007. (DOCX) [file pone.0106210.s004.docx]

**Appendix Table S4. Sample characteristics of ever-married women aged 20-24 years in Pakistan, 1991 and 2007.**

|  | 1991 | 2007 |
| --- | --- | --- |
|  | N=1064 | N=1560 |
|  | Weighted percentage (95% CI) | Weighted percentage (95% CI) |
| Age at marriage |  |  |
| <14 | 11% (8%-13%) | 5% (4%-6%) |
| 14-15 | 19% (16%-23%) | 18% (17%-20%) |
| 16-17 | 22% (19%-25%) | 26% (24%-29%) |
| ≥18 | 48% (44%-52%) | 50% (47%-52%) |
| Age at Interview |  |  |
| 20 | 30% (27%-34%) | 23% (20%-25%) |
| 21 | 10% (8%-13%) | 14% (12%-16%) |
| 22 | 26% (23%-29%) | 24% (22%-26%) |
| 23 | 19% (15%-22%) | 17% (15%-18%) |
| 24 | 15% (12%-18%) | 23% (20%-25%) |
| Education level |  |  |
| None | 75% (72%-79%) | 58% (55%-61%) |
| Any primary education | 13% (10%-15%) | 18% (16%-20%) |
| Any secondary education | 11% (9%-13%) | 19% (17%-21%) |
| Any higher education | 1% (1%-2%) | 5% (4%-6%) |
| Rural residence | 71% (68%-74%) | 70% (68%-72%) |
| Wealth quintile |  |  |
| Poorest | 20% (15%-24%) | 20% (17%-22%) |
| Poorer | 20% (16%-23%) | 23% (21%-25%) |
| Middle | 22% (19%-26%) | 20% (18%-22%) |
| Richer | 21% (17%-25%) | 19% (17%-21%) |
| Richest | 17% (14%-20%) | 18% (16%-20%) |
| Age gap† | 21% (18%-25%) | 19% (17%-21%) |
| Education gap *§* | 0.0 (-0.6-5.3) | 2.0 (-0.5-6.3) |

*†≥10 year age gap between husband and wife ± Median and IQR §Years of completed education of wife subtracted from years of completed education of husband; Median and IQR*
