# Supplementary material for: Cross-Sectional Time Series Analysis of Associations between Education and Girl Child Marriage in Bangladesh, India, Nepal and Pakistan, 1991-2011
Source: PLoS One. 2014 Sep 9;9(9):e106210. doi: 10.1371/journal.pone.0106210 (PMC4159189; doi:10.1371/journal.pone.0106210)
Supplement: Table S5 — Odds of girl child marriage in Bangladesh, India, Nepal and Pakistan by expanded education level. (DOCX) [file pone.0106210.s005.docx]

**Appendix Table S5. Odds of girl child marriage in Bangladesh, India, Nepal and Pakistan by expanded education level.**

|  | <14 years | 14-15 years | 16-17 years |
| --- | --- | --- | --- |
|  | AOR (95% CI) | AOR (95% CI) | AOR (95% CI) |
| **BANGLADESH** |  |  |  |
| **Education level** |  |  |  |
| No education | [REF] | [REF] | [REF] |
| Any primary education | 0.68 (0.47,1.00) | 0.76 (0.51,1.12) | 1.03 (0.66,1.60) |
| Secondary education |  |  |  |
| 0-1 completed years | 0.24 (0.12,0.50)*** | 0.47 (0.23,0.95) * | 1.13 (0.55,2.31) |
| 2 completed years | 0.32 (0.14,0.70) ** | 0.64 (0.31,1.32) | 1.54 (0.73,3.27) |
| 3 completed years | 0.09 (0.04,0.18) **** | 0.20 (0.11,0.36) **** | 0.51 (0.28,0.93) * |
| 4 completed years | 0.04 (0.02,0.08) **** | 0.20 (0.12,0.34) **** | 0.56 (0.34,0.94) * |
| ≥5 completed years | 0.01 (0.00,0.04) **** | 0.05 (0.02,0.11) **** | 0.30 (0.16,0.58) *** |
| Any higher education† | 0.00 (0.00,0.03) **** | 0.02 (0.01,0.05) **** | 0.21 (0.11,0.37) **** |
| **Time (survey year)** | 0.97 (0.92,1.03) | 1.04 (0.99,1.10) | 1.04 (0.98,1.10) |
| **Education gap§** | 0.97 (0.95,1.00) * | 0.97 (0.94,0.99) ** | 0.99 (0.96,1.01) |
| **INDIA** |  |  |  |
| **Education level** |  |  |  |
| No education | [REF] | [REF] | [REF] |
| Any primary education | 0.33 (0.27,0.40) **** | 0.50 (0.44,0.57) **** | 0.69 (0.61,0.78) **** |
| Secondary education |  |  |  |
| 0-1 completed years | 0.10 (0.07,0.15) **** | 0.33 (0.28,0.39) **** | 0.66 (0.56,0.77) **** |
| 2 completed years | 0.04 (0.03,0.08) **** | 0.19 (0.15,0.25) **** | 0.50 (0.40,0.62) **** |
| 3 completed years | 0.03 (0.02,0.06) **** | 0.15 (0.11,0.19) **** | 0.39 (0.32,0.48) **** |
| 4 completed years | 0.02 (0.01,0.07) **** | 0.08 (0.05,0.13) **** | 0.33 (0.25,0.44) **** |
| ≥5 completed years | 0.01 (0.00,0.04) **** | 0.05 (0.03,0.08) **** | 0.26 (0.20,0.33) **** |
| Any higher education† | 0.01 (0.00,0.06) **** | 0.02 (0.01,0.06) **** | 0.15 (0.11,0.20) **** |
| **Time (survey year)** | 0.99 (0.97,1.00) ** | 1.00 (0.99,1.01) | 1.01 (0.00,1.02) |
| **Education gap§** | 0.96 (0.95,0.97) **** | 0.98 (0.98,0.99) **** | 1.00 (0.99,1.01) |
| **NEPAL** |  |  |  |
| **Education level** |  |  |  |
| No education | [REF] | [REF] | [REF] |
| Any primary education | 0.41 (0.23,0.74) ** | 0.71 (0.51,0.99) * | 0.96 (0.73,1.25) |
| Secondary education |  |  |  |
| 0-1 completed years | 0.59 (0.15,2.30) | 0.65 (0.37,1.16) | 0.65 (0.37,1.15) |
| 2 completed years | 0.18 (0.06,0.52) ** | 0.43 (0.20,0.91) * | 1.05 (0.55,2.02) |
| 3 completed years | 0.60 (0.20,1.83) | 0.33 (0.14,0.77) * | 0.88 (0.45,1.70) |
| 4 completed years | 0.11 (0.03,0.43) ** | 0.08 (0.04,0.14) **** | 0.37 (0.22,0.61) *** |
| ≥5 completed years | 0.00 (0.00,0.07) *** | 0.06 (0.03,0.13) **** | 0.28 (0.17,0.45) **** |
| Any higher education† | 0.04 (0.00,0.53) * | 0.02 (0.00,0.13) **** | 0.09 (0.04,0.21) **** |
| **Time (survey year)** | 0.94 (0.91,0.98) ** | 0.99 (0.97,1.01) | 1.01 (0.99,1.03) |
| **Education gap§** | 0.98 (0.94,1.02) | 0.99 (0.97,1.02) | 0.98 (0.96,1.01) |
| **PAKISTAN^1^** |  |  |  |
| **Education level** |  |  |  |
| No education | [REF] | [REF] | [REF] |
| Any primary education | 0.67 (0.34,1.33) | 0.71 (0.38,1.34) | 0.95 (0.48,1.87) |
| Secondary education |  |  |  |
| 0-1 completed years | 0.98 (0.12,8.05) | 0.59 (0.12,3.01) | 0.07 (0.01,0.67) * |
| 2 completed years | 0.22 (0.02,1.96) | 0.45 (0.05,3.84) | 0.65 (0.19,2.26) |
| 3 completed years | 0.03 (0.00,0.22) *** | 0.10 (0.01,0.82) * | 0.78 (0.30,2.03) |
| 4 completed years | 1.27 (0.15,10.71) | 0.60 (0.08,4.29) | 0.00 (0.00,0.00) **** |
| ≥5 completed years | 0.02 (0.01,0.10) **** | 0.13 (0.03,0.62) * | 0.62 (0.25,1.54) |
| Any higher education† | 0.00 (0.00,0.00) **** | 0.00 (0.00,0.00) **** | 0.00 (0.00,0.00) **** |
| **Time (survey year)** | 0.96 (0.94,0.99) ** | 0.99 (0.97,1.01) | 1.02 (1.00,1.04) |
| **Education gap§** | 0.97 (0.93,1.01) | 1.00 (0.97,1.03) | 1.00 (0.98,1.03) |

*Adjusted for urban/rural residence, wealth quintile, 10+ year age gap between husband and wife, state of residence and education level by survey year interaction. Interaction effects were p<.1 for Bangladesh and Nepal, and p<.001 for India and Pakistan. †Higher education estimates may be inaccurate due to small cell sizes. §Years of completed education of wife subtracted from years of completed education of husband ^1^Unstable estimates were seen in the Pakistan analysis suggesting sample size was inadequate to yield interpretable findings. *p<0.05 **p<0.01 ***p<0.001 ****P<0.0001*
