# Supplementary material for: Cross-Sectional Time Series Analysis of Associations between Education and Girl Child Marriage in Bangladesh, India, Nepal and Pakistan, 1991-2011
Source: PLoS One. 2014 Sep 9;9(9):e106210. doi: 10.1371/journal.pone.0106210 (PMC4159189; doi:10.1371/journal.pone.0106210)
Supplement: Table S6 — Annual reductions in girl child marriages attributable to universal access to secondary education. (DOCX) [file pone.0106210.s006.docx]

**Appendix Table S6. Annual reductions in girl child marriages attributable to universal access to secondary education.** Based on 2012 probability estimates derived from married women aged 20-24 years for Bangladesh, India Nepal, and Pakistan.

|  | Bangladesh | India | Nepal | Pakistan | Overall |
| --- | --- | --- | --- | --- | --- |
| Proportion reduction in marriage <18 | 7.6% (5.3 to 9.8) | 6.3% (5.2 to 7.3) | 6.9% (3.9 to 9.9) | 5.5% (-0.3 to 11.4) | 6.5% (4.7 to 8.2) |
| Number of girls expected to marry <18 | 1,209,830 | 4,921,250 | 168,444 | 613,031 | 6,912,555 |
| Number of girls married <18, annual estimate* | 91,469 (64,145 to 118,793) | 309,473 (258,309 to 360,637) | 11,666 (6,609 to 16,723) | 33,960 (1,698 to 69,618) | 446,567 (327,364 to 565,771) |

*Based on 2012 population estimates^31^
